# How the COVID-19 Pandemic and its Consequences Affect the Presence of and Search for Meaning of Life: A Longitudinal Study

**DOI:** 10.1007/s10902-022-00592-5

**Published:** 2022-10-25

**Authors:** Rosa Mª Baños, Lorena Desdentado, Mª Dolores Vara, Tamara Escrivá-Martínez, Rocío Herrero, Marta Miragall, José M. Tomás

**Affiliations:** 1 Polibienestar Research Institute, University of Valencia, Valencia, Comunidad Valenciana Spain; 2 CIBER of Physiopathology of Obesity and Nutrition (CIBEROBN), Instituto de Salud Carlos III, Madrid, Spain; 3 Department of Personality, Evaluation, and Psychological Treatments, University of Valencia, Valencia, Spain; 4 Department of Psychology and Sociology, Universidad de Zaragoza, Teruel, Spain; 5 Department of Methodology for the Behavioral Sciences, University of Valencia, Valencia, Spain

**Keywords:** COVID-19 pandemic, Lockdown, Presence of meaning in life, Search for meaning in life, Latent growth models

## Abstract

The presence of meaning in life (PML) and the search for meaning in life (SML) are crucial when facing difficult times. Although several theoretical frameworks have tried to explain the dynamics of meaning in life during adversity, empirical evidence about interactions among both constructs using longitudinal designs is scarce. This study examined the trajectories of both PML and SML during the COVID-19 lockdown period in Spain. In total, 220 adults fulfilled an online survey during two periods: a strict and a relaxed lockdown period. Latent growth models showed that both PML and SML declined slightly during the strict lockdown, but they reached a plateau during the relaxed lockdown. Results also showed that age and having a partner predicted higher PML and lower SML at baseline, whereas being male predicted higher scores on PML. PML and SML were negatively associated at baseline, higher SML at baseline was related to a steeper decreasing PML slope during the strict lockdown, and the PML and SML slopes in the relaxed lockdown period were negatively related. This study contributes to better understanding longitudinal fluctuations of meaning in life in situations of adversity.

## Introduction

Meaning in life (MIL) has been defined as the extent to which people understand and find meaning in their lives, make sense of their lives (Steger et al., 2006), and perceive that they have a purpose, and it is a crucial ingredient of mental well-being and optimal functioning in times of crisis (Steger et al., 2006). This construct has received considerable attention from philosophers, theologians, and citizens from ancient times to the present day. Viktor E. Frankl postulated one of the most comprehensive theoretical conceptualizations of MIL more than 60 years ago (Frankl, 1959). He considered that human beings have as their primary intrinsic motivation to find meaning in their lives, being this possible to achieve even in extremely difficult circumstances. This conceptualization was initially operationalized by Crumbaugh and Maholick (1969/1981) and Crumbaugh (1977), who developed the two first questionnaires to measure it.

On March 11, 2020, the World Health Organization (2020) recognized the COVID-19 crisis as a global pandemic. From that date on, the number of infections and deaths grew exponentially worldwide. To curb the spread of the virus, the Spanish government declared a state of alarm on March 14th and implemented measures that included the closure of public spaces and home confinement, among others. Although these restrictions helped to reduce infection rates, they affected people's mental health and well-being (Valiente et al., 2021). More than a quarter of the population was affected by symptoms of depression, anxiety, and stress during the strict lockdown (Ozamiz-Etxebarria et al., 2020). Moreover, this symptomatology may be especially challenging because the COVID-19 pandemic is an unpredictable, long-lasting, and far-reaching event (Brooks et al., 2020).

Although it is crucial to investigate the negative impact of the pandemic on mental health, it is also necessary to examine how this challenging life event may offer the opportunity for growth through searching for and finding new MIL. How people responded to the COVID-19 pandemic could be related to the role played by the mortality salience felt, triggered by the belief that the virus is life-threatening (Pyszczynski et al., 2021). In this sense, the “terror management theory” (Greenberg et al., 1986) states that people manage the anxiety inherent to the awareness of death with proximal and distal defenses. These defenses construct a symbolic conception of reality that fills life with order, predictability, and significance, in which individuals obtain a sense of meaning in life and transcendence (Pyszczynski et al., 1999). During COVID-19, the saliency of death brought anxiety and fear, and these defenses wobbled impacting the previous conceptions of life and its meaning.

In recent decades, two different dimensions of MIL have been identified: the presence of meaning in life (PML) and the search for meaning in life (SML) (Steger et al., 2006). PML refers “to the degree to which people experience their lives as comprehensible and significant, and feel a sense of purpose or mission in their lives that transcends the mundane concerns of daily life”; whereas SML refers “to the dynamic, active effort people expend trying to establish and/or augment their comprehension of the meaning, significance, and purpose of their lives” (Steger et al., 2008a, 2008b, p. 661).

MIL may vary, among other factors, depending on the individual's developmental stage, gender, and marital status. People in later life stages report higher PML than younger people, who report higher SML (e.g., Steger et al., 2009). However, there is limited evidence about differences between men and women. Steger et al. (2009) showed that women tended to report higher PML and SML, but the differences did not reach significance when considering the effect of age. Similarly, Yu et al. (2017) also found no gender differences in mean scores on PML and SML. Regarding marital status, being married and living with a partner have both been proposed as buffers against a crisis of meaning (Schnell, 2009). Moreover, Damásio and Koller (2015) found that single people had lower levels of PML compared to other conditions (e.g., people in a stable relationship).

MIL can be affected by experiencing adverse events. Specifically, PML can decrease when experiencing stressors due to emerging discrepancies between previous cognitive models of the world (e.g., global beliefs, goals, and the subjective sense of meaning) and new information derived from the stressor, including the particular meaning assigned to the traumatic event (Park, 2010). “Meaning making*”* (that is, SML) is considered crucial in reducing these discrepancies and successfully adjusting to adversity (Gillies & Neimeyer, 2006). According to this approach, SML attempts are expected to decrease as PML is achieved, but continued SML is expected as long as meaning is not found (Park, 2010).

To date, most of the research has focused on analyzing the relationship between both PML and SML, and mental health outcomes (Li et al., 2021). However, there is increasing interest in the way PML and SML interact. Specifically, two models have been proposed to explain this interaction (Steger et al., 2008a, 2008b), which are complementary to the *meaning making model* (Park, 2010). On the one hand, according to the *presence-to-search* model, people who already have a sense of PML experience SML to a lesser extent; conversely, when the individual lacks PML, SML is expected to be high. On the other hand, according to the *search-to-presence* model, SML should lead to subsequently finding more PML (Steger et al., 2008a, 2008b). Steger et al. (2008a, 2008b) conducted a cross-sectional study that provided clear support for the *presence-to-search* model, but mixed support for the *search-to-presence* model*.* However, the nature of the relationship between SML and PML predicted by the *search-to-presence* model might be better suited to longitudinal designs than to cross-sectional designs. Two longitudinal studies found that baseline SML scores did not predict PML at one-year follow-up in undergraduate students (Steger & Kashdan, 2007) and chronic pain patients (Dezutter et al., 2015). In contrast, a longitudinal study using the daily diary method found that SML positively predicted PML the day after (Newman et al., 2017). Along these lines, Chu and Fung (2021) recently found that SML at baseline positively predicted PML at one-month and six-month follow-ups, but only in those with greater maladaptive traits (e.g., high avoidance orientation, low optimism).

These mixed findings highlight the possible influence of other factors, such as culture (Steger et al., 2008a, 2008b), personality variables, or psychological needs (Chu & Fung, 2021), on the directionality and strength of the relationship between PML and SML. Moreover, Chu and Fung (2021) highlighted that SML could involve different opposite connotations -which makes it even more difficult to understand these relationships-: “growth search” and “deficiency search”. On the one hand, “growth search*”* consists of attempting to deepen and further understand MIL under positive conditions (i.e., having a fulfilling PML). On the other hand, “deficiency search*”* is a struggle for people who have a meaning void, and it is characterized by trying to reduce the tension between their PML expectations and deficiency under negative conditions (i.e., not having a clear sense of PML or having a threatening PML) (Chu & Fung, 2021). Whereas a growth search might be pleasurable, a deficiency search might be distressing in the short term but later lead to higher PML when recovering from traumatic events.

This notion that SML at baseline predicts the development of PML at a follow-up was tested by these authors in Chinese undergraduate students (Chu & Fung, 2021). However, it is unknown whether this prediction can be generalized to other populations and in a context of adversity. In this regard, the COVID-19 pandemic has created an unprecedented opportunity to examine psychological processes in times of strife, as shown by the large number of studies recently published in this field (Odone et al., 2020). Examining the trajectories of PML and SML during different phases of the COVID-19 lockdown (such as the strict lockdown period and the subsequent relaxed lockdown period) would shed light on the dynamics of PML and SML when facing a chronic stressor that affects the whole world.

The general aim of this study is to explore trajectories of MIL throughout the lockdown due to the COVID-19 pandemic. The PML and SML of a sample of Spanish citizens were assessed every 2–3 days from March 27 to May 17, 2020, with a total of 23 measurements or waves of data collection. The first specific objective is to examine independent growth curves of PML and SML during the strict lockdown period (from March 27 to April 26) and the more relaxed lockdown period (from April 28 to May 17). The second specific objective is to determine whether some sociodemographic variables (age, gender, and having a partner or not) predicted different trajectories of both PML and SML, and whether baseline levels of SML predicted the PML trajectory and vice versa (i.e., whether baseline levels of PML predicted the SML trajectory). Finally, the third specific objective is to explore the relationships between trajectories of PML and SML during the lockdown.

First, drawing on the *meaning making model*, the first hypothesis is that PML will decrease and SML will increase during the strict COVID-19 lockdown, whereas the reverse trend is expected during the relaxed lockdown (i.e., PML will increase and SML will decrease when the lockdown is relaxed). The second hypothesis is that older individuals (vs. younger ones) and those with a partner (vs. singles) will report higher baseline levels of PML and lower levels of SML, whereas no differences between genders are expected. Given the novel nature of this study, no specific hypotheses were proposed about the relationship between these variables and the PML and SML curves during the lockdown. Third, based on the *search-to-presence model*, we hypothesize that SML at baseline will predict trajectories of increasing PML during the lockdown period. Conversely, based on the *presence-to-search* model, we expect that PML at baseline will not predict trajectories of SML during the lockdown. Instead, based on the *presence-to-search model*, we hypothesize that: (i) PML will be negatively associated with SML at baseline; and (ii) trajectories of PML and SML will covary negatively with each other, so that where one is upward, the other is downward.

## Method

### Participants

The study sample consisted of 220 participants aged between 18 and 68 years old (*M* = 36.04; *SD* = 13.54) who provided data in at least 50% of the 23 assessment waves (i.e., 12 or more). Of them, 82.73% were women, and 63.2% had a partner. Inclusion criteria were: 1) age ≥ 18 years and 2) living in Spain during the lockdown. On average, participants completed 18.86 of the 23 data collection waves (*SD* = 3.29). The sociodemographic characteristics of the sample are described in Table 1.

**Table 1:** Demographic characteristics of the sample and meaning in life at baseline

	*N* = 220
*Sex (%women)*	82.7%
*Age (years) M (SD)*	36.04 (13.54)
18–24 years old 25–35 years old 36–50 years old > 50 years old	22.7% 35.9% 22.7% 18.6%
*Marital status* Single In a relationship Married Divorced/separated Widowed Other	26.4% 38.2% 25.0% 7.3% 1.4% 1.8%
*Monetary income* Below the mean At the mean Above the mean	35.5% 52.3% 12.3%
*Employment situation* Employee (permanent job) Employee (temporal job) Freelancer Job seeker Student Other	32.7% 18.6% 3.6% 7.7% 24.5% 12.7%
*MLQ-Presence M (SD)* *MLQ-Search M (SD)*	25.17 (6.72) 18.74 (8.27)

MLQ = Meaning in Life Questionnaire

The study was performed following the ethical standards of the 1964 Declaration of Helsinki and approved by the ethics committee of the University of Valencia (Spain) (register number: 1593681212393). All participants agreed to participate and signed an online informed consent prior to their participation in the study. Subjects who signed up to participate in the study were entered into a drawing for 1 out of 10 Amazon gift cards valued at 40€ each to encourage participation.

### Procedure

Participants were recruited through social networks (WhatsApp, Facebook, Twitter, and Instagram). Data were collected through online surveys using the Qualtrics web platform. This longitudinal study began on March 27, 2020, during the COVID-19 pandemic in Spain (when the strictest lockdown since the pandemic began was taking place), and it ended on May 17, 2020 (when substantial relaxation of lockdown restrictions began). Volunteers were asked to complete the measures on Tuesdays, Fridays, and Sundays during this time span. As a result, 23 repeated measures were collected. Two periods of time can be distinguished based on the restrictive measures implemented. On the one hand, the first period was characterized by strict confinement measures and halting of “non-essential” work activities (unless they could be performed through teleworking), and it lasted until April 26 (Period 1). On the other hand, the second period was characterized by progressive relaxation of these restrictive measures and lasted from April 28 to the end (Period 2). Indeed, this second stage was called the "transition to a new normality" by the Spanish Government and Ministry of Health (2020).

### Measures

#### Sociodemographic Characteristics

Personal data included age, sex, marital status, monetary income, and employment situation.

#### Meaning in Life (Baseline Measurement)

The Meaning in Life Questionnaire (MLQ; Steger et al., 2006) was used to measure the predispositional MIL conceptualization as follows: (1) PML, that is, the extent to which participants feel their lives are meaningful (e.g., “I have a good sense of what makes my life meaningful”; MLQ-Presence); and (2) SML, that is, the extent to which they actively seek meaning in their lives (e.g., “I am always searching for something that makes my life feel significant”; MLQ-Search). The MLQ consists of 10 items rated on a 7-point Likert scale ranging from 1 (absolutely untrue) to 7 (absolutely true). Specifically, participants were asked to take a moment to think about what makes them feel that their life is important and meaningful. In this study, internal consistency was adequate for both the MLQ-Presence (α = 0.92) and MLQ-Search (α = 0.94) subscales.

#### Meaning in Life (Longitudinal Measurement)

As in previous work that included repeated measures using online tools (Suso-Ribera et al., 2018), two *ad-hoc* items were developed to longitudinally assess state-like MIL based on the MLQ (Steger et al., 2006). On the one hand, a visual analogue scale referred to PML (“At this moment, how much do you feel that your life has meaning?”). Item responses were scored from 0 (“My life has no meaning at all”) to 100 (“My life is full of meaning”). On the other hand, another visual analogue scale was used to measure SML (“At this moment, to what extent are you looking for something that makes you feel like your life has meaning?”), ranging from 0 (“I'm not looking for anything at all”) to 100 (“I'm constantly looking for something”).

### Statistical Analysis

First, SPSS 26.0 was used for data management and descriptive statistics. Second, Latent Growth Models (LGM) were specified and tested to analyse the trajectories of the PML and SML over time. LGMs are fit as restricted common factor models with latent variables for the intercept -which models the initial state-, and the slope -which models the rate of change or trajectory- (Grimm et al., 2017). The LGMs employed all the available time points in the analyses (i.e., 20 for PML and 23 for SML),^1^ and they were estimated in Mplus 8.4 (Muthén & Muthén, 1998–2017) with robust full information maximum likelihood estimation (MLR) to properly deal with missing data and the non-normal characteristics of the data. Several statistics and indexes were used to assess model fit. Hu and Bentler's (1999) recommendations for considering adequate fit were followed. Specifically, we used the Satorra–Bentler corrected chi-square statistic, the Comparative Fit Index (CFI), the standardized root-mean-square residual (SRMR), and the root mean square error of approximation (RMSEA) with a 90% confidence interval. Regarding the cut-off criteria, a model was considered to have a good fit if the CFI had a value of at least 0.90 (better if 0.95), a value close to 0.08 for SRMR, and a value close to 0.06 for RMSEA. P-values less than 0.05 were considered statistically significant.

## Results

### Unconditional Latent Growth Models

The trajectories of PML and SML across the time points were first studied with unconditional LGM. However, before estimating these models, the intraclass correlation coefficients (ICC) for both PML and SML were calculated. The results were 0.85 for PML and 0.83 for SML. These large values indicated that most of the variance was between subjects. Unconditional LGMs examine the growth trajectories of the response variable without covariates (Wang & Wang, 2020). These models try to estimate the function that best represents the shape of the longitudinal change. For each variable, two unconditional models were tested: (a) *Unconditional LGM with intercept but no slope* (inter-individual variability at the initial state but no tendency associated with time), which also serves as a baseline model; and (b) *a non-linear Piecewise LGM*. The Piecewise LGM is a semiparametric method that breaks the growth into separate linear segments or pieces, which is particularly interesting in our situation because of the two different periods during the lockdown depending on the restrictive measures adopted (see Procedure section) (Wang & Wang, 2020). Therefore, Slope 1 included the time points of the Period 1, and Slope 2 included the time points of the Period 2.

**Fig. 1:**
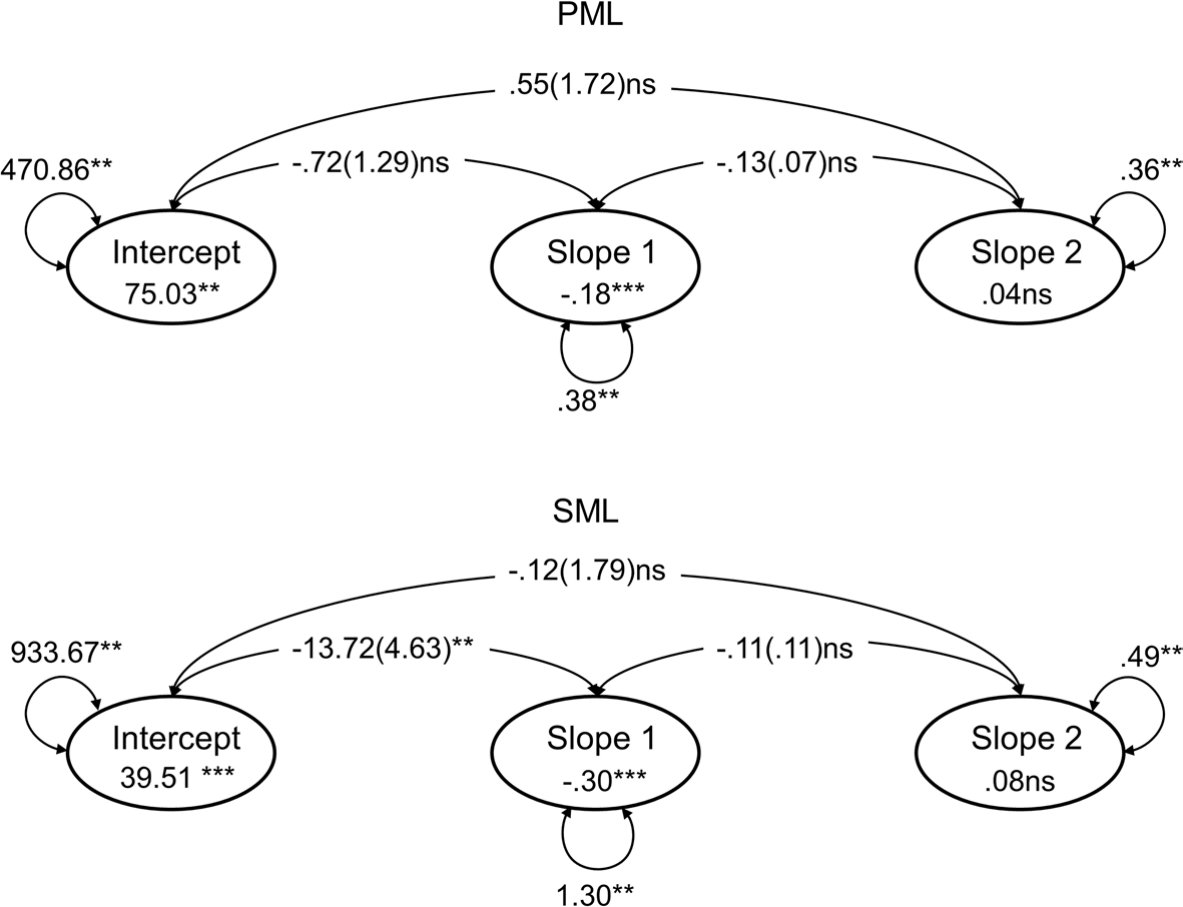
Unconditional Piecewise LGMs for PML and SML. *Note.* Intercepts represent the estimated initial state of trajectories of both PML and SML, respectively. Slopes 1 represent estimated trajectories of both PML and SML for Period 1 (between March 27, 2020 and April 26, 2020). Slopes 2 represent estimated trajectories of both PML and SML for Period 2 (from between April 28, 2020 and May 17, 2020). Parameter estimates (standard errors); double-headed curved arrows pointing to different latent variables show their covariances; double-headed curved arrows pointing from a latent variable to itself show variance estimates, and values in the circles represent unstandardized parameter estimates for the intercepts and slopes. *p < .05, ** *p* < .01, *** *p* < .001; *ns =* non-significant (*p* > .05)

Both the intercept only and piecewise models fitted the data well for PML (see Table 2). However, the piecewise model clearly improved the model fit, and therefore it can be considered a better representation of the data. The unstandardized estimates of the piecewise model for PML are graphically presented in Fig. 1. The mean intercept (estimated mean in Time 1) was 75.02 (in a range from 0 to 100), indicating that the sample started the lockdown relatively high in PML. The estimated slope for Period 1 indicated that for one unit of time (i.e., two days) change, the PML in the sample on average significantly decreased by 0.174 points (standardized estimate: *β* =−0.202, *p* < 0.001). On the other hand, the slope for Period 2 was not statistically significant, indicating that the loss of PML reached a plateau when the lockdown was relaxed (*β* = 0.067, *p* > 0.05), which was at a mean score of 72.61. These estimates showed that there was statistically significant variability in the intercept (i.e., initial time point or baseline), in the slope during the strict lockdown (Period 1), and in the slope during the relaxed lockdown (Period 2). The figure also shows that none of the relationships between the intercept, Slope 1, and Slope 2 were statistically significant, indicating that the initial point for PML is not related to a specific trend in the PML Slopes 1 and 2, and that these are not associated with each other.

**Table 2:** Fit indexes for the sequence of latent growth models for PML and SML

Latent Growth Model (LGM)	*χ*^2^	df	*p*	RMSEA	CI 90%	CFI	SRMR
*PML*							
Intercept only LGM	489.1	208	< .01	.078	.069–.087	.921	.058
Piecewise LGM	308.7	201	< .01	.049	.038–.060	.970	.037
Conditional LGM	399.4	269	< .01	.047	.037–.057	.969	.034
Final Conditional LGM	410.2	279	< .01	.046	.036–.056	.969	.035
*SML*							
Intercept only LGM	837.3	274	< .01	.095	.089–.104	.845	.055
Piecewise LGM	389.0	267	< .01	.046	.035–.055	.967	.029
Conditional LGM	492.7	347	< .01	.044	.035–.053	.966	.028
Final Conditional LGM	496.4	335	< .01	.047	.038–.056	.961	.092
*Parallel LGMs*							
Parallel LGM	1811.1	919	< .01	.066	.062–.071	.939	.037
Final Parallel LGM	1815.9	928	< .01	.066	.061–.070	.939	.038

*LGM = *Latent growth model. *χ*^2^ = Satorra−Bentler corrected Chi−Square statistic. *df* = degrees of freedom. *RMSEA* = Root mean squared error of approximation. *RMSEA* 90% *CI* = Root mean squared error of approximation with a 90% confidence interval. *CFI = * Comparative Fit Index. *SRMR* = Standardized root−mean−square residual

**Fig. 2:**
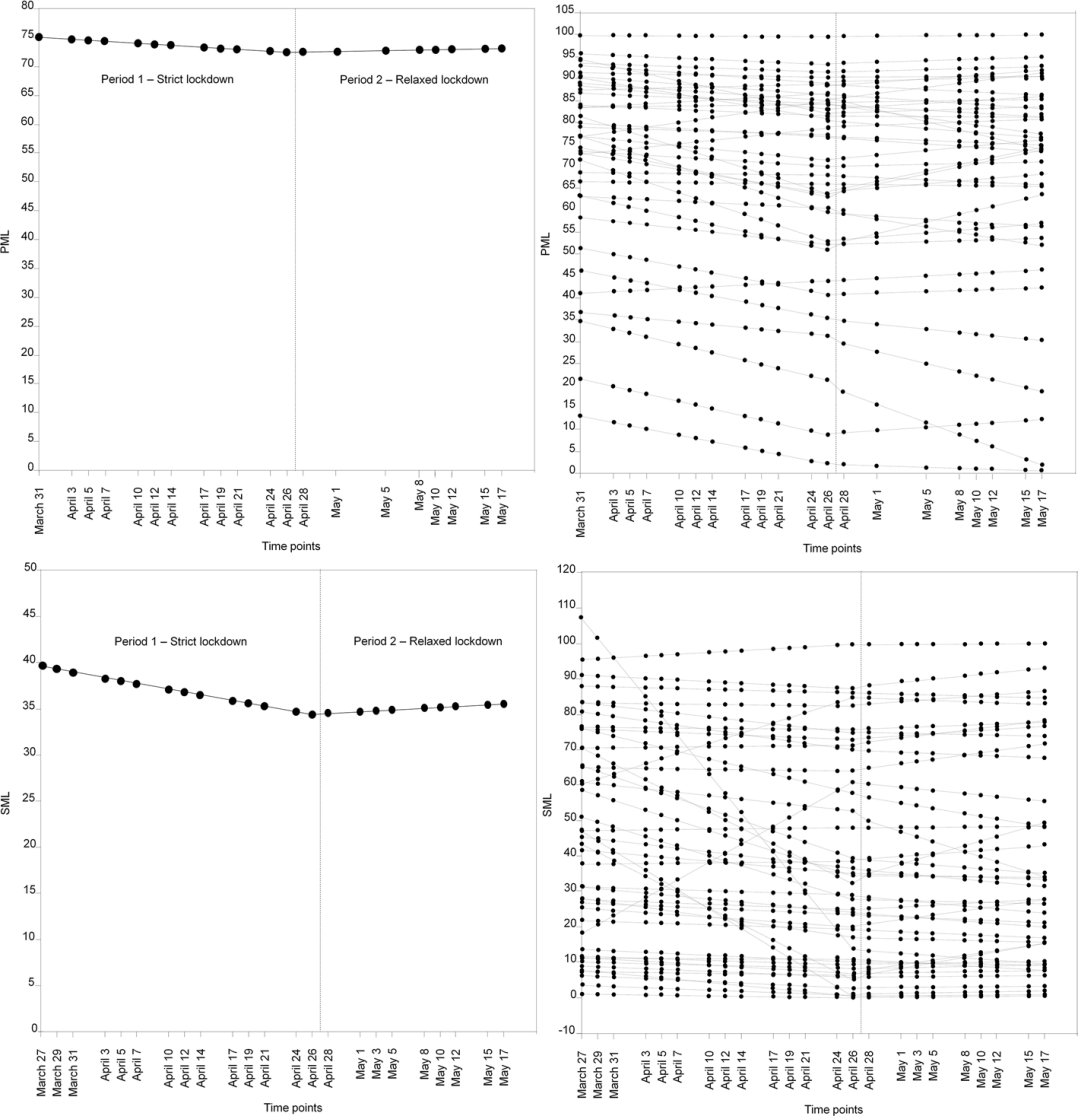
Estimated trajectories for the total sample (left column) in both PML and SML and 50 random individual trajectories (right column). *Note.* Scores on PML and SML were measured using *ad-hoc* visual analogue scales ranging from 0 to 100. The dashed vertical line (between April 26 and April 28) represents the transition point between the two periods

With regard to SML, again, both models (intercept only and piecewise) fitted the data well, but the piecewise model greatly improved the fit. The unstandardized estimates of the piecewise model for SML are graphically presented in Fig. 1. The mean intercept was 39.49, indicating that the sample started the lockdown relatively low in SML. The estimated slope during the strict lockdown (Period 1) indicated that for one unit of time (i.e., two days) change, the SML in the sample, on average, significantly decreased by 0.301 points (standardized estimate: *β* = -0.264, *p* < 0.001). However, as occurred with PML, the slope during the relaxed lockdown (Period 2) was not statistically significant, indicating that the loss of SML reached a plateau when the lockdown restrictions were relaxed (*β* = 0.122, *p* > 0.05), which was at a mean score of 35.34. These estimates show that there is statistically significant variability in the intercept, in Slope 1, and in Slope 2. The figure also shows that the intercept is negatively related to Slope 1, which indicates that those who started with higher levels of SML had a larger decrease in SML during the strict lockdown. Finally, there were no significant associations between the intercept and Slope 2 or between Slopes 1 and 2. Figure 2 shows the estimated PML and SML trajectories for the total sample and 50 random individual trajectories.

### Conditional Latent Growth Models

First, we estimated a conditional LGM of the intercept and trajectories of PML, with the following predictors: age, gender, marital status, and MLQ-search. Table 2 shows goodness-of-fit indices for this a priori conditional model. Although the model fit was adequate, there were some non-significant coefficients. Specifically, the predictors had no significant effects on either Slope 1 and Slope 2 for PML, whereas they had significant effects on the PML intercept. Therefore, the statistically non-significant effects were removed, and a new model, also good-fitting, was estimated (see Table 2). This model showed that men started out, on average, 7.16 points higher on PML than women (*β* = 0.128, *p* < 0.05). Regarding the effect of marital status, people with a partner had an average PML 7.30 points higher than those without a partner at Time 1 (*b* = -7.301, *β* = -0.163, *p* < 0.05). Age was positively related to PML at baseline. Specifically, a one-year increase in age was associated with 0.206 more points on PML (*β* = 0.165, *p* < 0.05). Finally, MLQ-Search was negatively related to the PML intercept, which means that the less SML people had at baseline, the more PML they had (*b* = -0.61, *β* = -0.23, *p* < 0.05).

Second, a conditional model was estimated to predict the intercept and the two trajectories of SML. The predictors considered were age, gender, marital status, and MLQ-Presence. Table 2 shows that an initial conditional model fitted the data well. However, the only significant effects on the intercept were produced by marital status, age, and MLQ-Presence. No other effects were statistically significant. Therefore, a second conditional (final) model was estimated with only the significant effects, and this model also fitted the data well (see Table 2). The effect of marital status was positive (*b* = 10.61, *β* = 0.175, *p* < 0.05), indicating that people without a partner scored 10.61 points higher on SML than people with a partner, on average. However, age and MLQ-Presence were negatively related to the intercept of SML: age, *b* = -0.42, *β* = -0.19, *p* < 0.05; MLQ-Presence, *b* = -0.81, *β* = -0.19, *p* < 0.05. Specifically, a one-year decrease in age was associated with 0.42 more points on SML baseline, and a one-point decrease in MLQ-Presence was associated with 0.81 more points on SML baseline.

### Parallel Latent Growth Models

Finally, we estimated a parallel LGM in which the repeated measures for both PML and SML are modelled together in order to study the associations between their respective intercepts and slopes (Wang & Wang, 2020). The goodness-of-fit indices in Table 2 show that this parallel model fitted the data well. However, some of the associations between intercepts and slopes were not statistically significant. Therefore, a simpler model with only the statistically significant covariances was estimated. It had a good fit and is presented here. The main results are: (1) both intercepts were statistically related, and the association was negative, indicating that those who scored high on PML tended to be low on SML at baseline (*r* = -0.253, *p* < 0.05); (2) there was a significant and negative correlation between the intercept of SML and the negative trajectory of PML (Slope 1) (*r* = -0.186, *p* < 0.05). This result indicates that those with a higher initial score on SML had a more negative PML trajectory during the strict lockdown (i.e., a larger decrease of PML during Period 1); and (3) the PML and SML slopes for Period 2 were also negatively related (*r* = -0.355, *p* < 0.05).

## Discussion

The aim of this study was to explore the trajectories of both PML and SML during the COVID-19 lockdown in Spain. Specifically, two distinct lockdown periods (including three assessment waves per week) were analyzed: the strictest lockdown since COVID-19 began (Period 1); and the subsequent lockdown period, characterized by a progressive easing of restrictive measures (Period 2).

First, results show that both PML and SML significantly decreased during the strict COVID-19 lockdown (Period 1). However, they did not continue to decrease; instead, they stabilized when restrictive lockdown measures began to be relaxed (Period 2). Hence, our results partially supported our first hypothesis. The decrease in PML during the strict lockdown (Period 1) is in line with previous research indicating that current suffering is associated with a reduction in the sense of MIL. This finding has been found regardless of the specific event (Edwards & Van Tongeren, 2020), but also associated with COVID-19 suffering (Arslan et al., 2020). Moreover, Edwards and Van Tongeren (2020) also found that the time since the experience of suffering was positively associated with PML. In other words, past suffering (compared to current suffering) has the potential to promote PML, which, in turn, is closely related to post-traumatic growth and life satisfaction (Triplett et al., 2012). Although PML is considered a trait-like protective factor against the impact of negative life events, including the COVID-19 pandemic (Humphrey & Vari, 2021), this is not incompatible with its weakening when going through a difficult time and its ability to increase again when the adverse event has been overcome. In this regard, not finding a significant increase in PML during the more relaxed lockdown (Period 2) could be explained by at least the following two considerations. First, the relaxed lockdown (Period 2) was still a lockdown (including home confinement for most people and physical and social distancing) instead of a so-called “new normality”, and, therefore, the stressor had not disappeared yet. Indeed, the risk and danger of infection, disease, and death caused by the virus persisted. Second, the temporal proximity of the strict lockdown (Period 1) was close, so that not enough time had passed to re-make meaning. According to the Terror Management Theory, situations in which people face death impact the way they view life and its meaning (Pyszczynski et al., 1999). Therefore, the recovery from this situation and rebuilding of a new sense of meaning might take longer.

Contrary to what was hypothesized, SML seems to decrease rather than increase during the strict COVID-19 lockdown. Previous research on SML in the context of trauma and loss has shown that people who are facing adversity are more likely to search for meaning, which could be a critical mechanism in making sense of a traumatic event and, in turn, achieving successful psychological adjustment (e.g., Davis et al., 2000). Again, the period of time during which MIL was repeatedly assessed in this study (i.e., the lockdown) might not have been long enough to capture an overall meaning-making process -which refers to efforts to reduce the discrepancy between the appraised meaning of the current pandemic and pre-existing global beliefs (Park, 2010)-. In this regard, studies on the period of time during which the meaning-making process occurs have shown heterogeneous results, ranging from the first weeks to several years after a traumatic event (Davis et al., 2000; Murphy et al., 2003). Therefore, further research is needed to clarify the temporal course of the activation of SML and its determinants during and after adversity. Moreover, according to our results, those individuals with higher SML at baseline experienced a larger decrease in SML during the strict lockdown (Period 1). It might be that these individuals were reactive early in lockdown, showing higher levels of SML at baseline, and their greater decline in this variable throughout the strict lockdown may reflect a return to a baseline similar to that of the rest of the sample.

Another explanation for the overall decrease in SML is related to the unclear nature of the pandemic as a long-term stressor from the beginning. For instance, people initially thought the lockdown would not last more than 15 days, and they gradually realized that the situation was going to drag on. Thus, the changing expectations produced by this unprecedented situation might have played a relevant role in the unexpected decrease in SML during the strict lockdown. In addition, the decrease in SML could also be influenced by the type of stressor, which was not individual but universal, affecting everyone. Specifically, the restriction measures were global and had to be followed by everyone because the risk of infection and disease was shared. The fact that a stressor is not just “personal” may initially trigger less search for meaning. Future studies should explore whether different types of stressors with distinct situational and global determinants have different effects on the meaning-making process.

Additionally, this study also revealed significant individual differences in the baseline PML and SML scores based on sociodemographic variables, but not in the slopes throughout the lockdown. Our hypotheses in this regard were partially supported. First, men unexpectedly reported higher initial scores on PML than women, whereas there was no significant effect of gender on SML. Previous research has shown mixed results about differences in MIL based on gender. Whereas some studies reported higher PML and SML in women than in men (Steptoe & Fancourt, 2019), other studies found no gender differences (Steger et al., 2009; Yu et al., 2017). It should be noted that our results could be biased because the male gender was underrepresented in our sample. Further research is needed to better understand MIL from a gender perspective, especially because the pandemic has exacerbated existing gender inequalities (e.g., mothers reported a greater increase in household and care work than fathers) (Carlson et al., 2020; Fisher & Ryan, 2021), which could also affect the dynamics of MIL.

Second, our results showed that age was a significant predictor of higher scores on PML and lower scores on SML at baseline. These results are consistent with previous studies (Steger et al., 2009) and with developmental theories suggesting that SML is expected to be higher in earlier life stages, such as emerging adulthood, than in later stages (e.g., Erikson, 1968). However, there is also existing evidence indicating a small age-related decline in PML, which is stronger in older people (Irving et al., 2017). As Steger et al. (2009) pointed out, the measure used in different studies has an influence on age-related changes in PML levels. Specifically, studies that reveal a decrease in PML associated with increasing age are characterized by including PML measures that focus on usefulness and setting goals, rather than on the sense of a meaningful life.

Third, individuals with a partner had higher scores on PML and lower scores on SML at baseline than single people, which is also consistent with our initial hypothesis. Loneliness has been shown to be associated with low PML (Macià et al., 2021; Stillman et al., 2009). Although the conceptualization of loneliness is a subjective experience that involves being apart from others, rather than a lack of companionship (Bekhet et al., 2008), the physical isolation due to the lockdown could equate this experience -especially for people who lived alone and/or did not have enough technological skills or resources to communicate with their loved ones-.

With regard to the hypotheses based on the *search-to-presence model* (Steger et al., 2008a, 2008b), contrary to what was expected, we found that scores on SML at baseline (i.e., intercept) were negatively related to the *negative* trajectory of PML during the strict lockdown (Period 1), whereas a non-significant relationship was found with the PML trajectory during the relaxed lockdown (Period 2). However, it should be noted that this pattern was not observed in the conditional LGM for PML trajectories regressed on the MLQ-Search scores. These findings indicate that being involved in state-like SML at the beginning of the lockdown (rather than showing high dispositional SML measured with the MLQ) was related to a greater decrease of PML in Period 1. This result could suggest that individuals who did not initially have a sufficiently robust meaning frame to cope with the pandemic situation were even more vulnerable to loss of meaning over the strict lockdown. Again, more longitudinal evidence over a more extended time (e.g., beyond the end or relief of the stressful burden) is needed to provide insights into how, when, and through what mechanisms or determinants SML leads to building PML when facing adversity.

Regarding our last hypothesis, results revealed that PML and SML showed a significant negative relationship, which was confirmed both cross-sectionally and longitudinally. On the one hand, conditional LGMs showed that baseline scores on MLQ-Search were significantly associated with lower scores on PML baseline (i.e., intercept of the PML trajectory), whereas baseline scores on MLQ-Presence were significantly associated with lower scores on SML baseline (i.e., intercept of the SML trajectory). Similarly, findings from parallel LGMs showed that baseline levels of both PML and SML were negatively related. On the other hand, relaxed-lockdown trajectories (Period 2) of PML and SML negatively covaried with each other. This could suggest that those with a positive PML slope tended to have a negative SML slope (and the other way around) when restrictive measures were easing. However, given that these slopes were not significantly different from zero, it is also possible that those with a positive PML slope tended to have a non-significant SML slope as opposed to a negative SML slope. Future studies should determine how steep increases in SML (vs. steep drops in SML) are related to the trajectory of PML and vice versa.

These findings support the *presence-to-search model* (Steger et al., 2008a, 2008b), indicating that individuals with higher PML simultaneously show lower SML and vice versa, even in adverse situations such as the beginning of the COVID-19 pandemic. In other words, individuals who search for MIL experience lower PML, whereas individuals who already have PML search less for MIL. Moreover, gaining PML during the relaxed lockdown was negatively related to the trajectory of SML, that is, those who were finding meaning seemed to stop seeking or search for it less than those who experienced less meaning. To the best of our knowledge, this is the first study to provide longitudinal evidence for this theoretical model. However, it should be noted that this trend was not found during the strict lockdown (Period 1). A possible explanation could be that other variables, such as individual perceived stress, the specific type of stressor, or uncertainty, might be affecting not only the PML and/or SML trajectories, but also the relationship between PML and SML throughout the pandemic. In this line, a recent study showed that intolerance of uncertainty moderated the effects of SML on PML because individuals with high (vs. low) intolerance of uncertainty reported less PML when engaging in SML (Morse et al., 2021). Therefore, these potential moderators should be considered when examining PML and/or SML in traumatic events.

This study has relevant theoretical and practical implications. Theoretically, this study broadens insights about MIL conceptualized as a “state” (Chu & Fung, 2021; Newman et al., 2017), which should be understood as complementary to approaches conceptualizing MIL as a stable trait. Ultimately, the evidence derived from this work contributes to the theoretical knowledge about how PML and SML interact and evolve during a prolonged stressor characterized by physical isolation and high uncertainty, as well as during its progressive easing. To our knowledge, this is the first study to longitudinally explore the course of both PML and SML in the context of COVID-19. Future research should examine the generalizability of these findings by exploring MIL in a wide range of chronic stressful events. Moreover, studies should also explore whether specific MIL patterns contribute to better coping and growth following adversity. Regarding the practical implications, the present study suggests that psychological interventions designed to promote mental health in the general population during the COVID-19 pandemic should consider that strict lockdowns might involve decreasing PML and SML, especially when there is no estimated end date. In this line, a recent study found positive effects of a “meaning salience” intervention (i.e., intervention aimed at increasing awareness of one’s MIL) on anxiety, depression, and stress over a week during the COVID-19 pandemic (April 2020) (Klussman et al., 2021).

Several limitations of this study should be considered. First, although it included numerous repeated measures for almost two months in a critical period of the COVID-19 pandemic, this study does not shed light on the course of MIL when the lockdown was completely over (i.e., when the state of alarm expired on June 21^st^, 2020). More extended longitudinal studies are needed to clarify not only the fluctuations in PML and SML during distressing situations such as a lockdown, but also beyond them, that is, once they have ended and a “new normality” is established. Second, a pre-pandemic baseline measure of PML and SML is not available because the first data collection (which also included the MLQ questionnaire) was performed after the lockdown had already started. Therefore, our results do not allow us to fully explore the role of MIL as “trait” in MIL as “state” when going through difficult situations. Third, our sample showed particularly high scores on PML (mean scores around 75) and low scores on SML (mean scores around 39) at baseline, which could affect our results. Finally, the sample was not particularly large, although the inclusion of many time points may compensate for statistical power by contributing more observed information to the estimation of the LGMs (Muthén & Curran, 1997).

In conclusion, this study contributes to better understanding the dynamic process of MIL during a lockdown. In particular, these results provide longitudinal evidence for the most influential models of the trajectory and interaction between PML and SML in the initial period of COVID-19. Overall, both PML and SML decreased during the strict lockdown, but they became stable when the lockdown was easing. The baseline scores of both trajectories varied depending on whether they had a partner and their age, whereas baseline PML also varied based on gender. Finally, baseline SML predicted a more negative PML slope during the strict lockdown, and PML and SML showed negative associations at baseline and during the relaxed lockdown, supporting the presence-to-search model.

## Footnotes

1 Three repeated measures of PML were missing due to technical issues, namely, March 27, March 29, and May 3.
